# Supplementary material for: Changes in the Choroidal Thickness following Intravitreal Bevacizumab Injection in Chronic Central Serous Chorioretinopathy
Source: J Clin Med. 2022 Jun 13;11(12):3375. doi: 10.3390/jcm11123375 (PMC9224583; doi:10.3390/jcm11123375)
Supplement: Supplementary file 1 [file jcm-11-03375-s001.zip › jcm-1718591-supplementary.pdf]

Table S1. Changes in the Choroidal Thickness following Intravitreal Bevacizumab Injection in Chronic Central Serous Chorioretinopathy

Yoo-Ri Chung, Su Jeong Lee, Ji Hun Song
Department of Ophthalmology, Ajou University School of Medicine, Suwon, Republic of Korea

Corresponding author: Ji Hun Song, MD, PhD
Department of Ophthalmology, Ajou University School of Medicine, 164 World Cup-ro, Yeongtong-gu, 16499 Suwon, Republic of Korea

Included eye	Age (years)	Sex (1:M, 2:F)	HTN (0: no, 1: yes)	Smoking (0:no, 1: yes)	Group (1: Refractory, 2: Resolved)	FA leakage (0: no, 1: yes)	Pre VA	Pre VA (logMAR)	Pre CRT (μm)	Pre SFCT (μm)	1Mo VA	1Mo VA (logMAR)	1Mo CRT (μm)	1Mo SFCT (μm)	Last VA	Last VA (logMAR)	Last CRT (μm)	Last SFCT (μm)	F/U period (months)	No. of IVB for resolution	Total No. of IVB
1	49	1	0	1	2	1	0.32	0.4949	689	484	0.5	0.301	168	403	0.63	0.2007	206	402	30	1	5
2	51	2	0	0	2	0	0.2	0.699	417	228	0.25	0.6021	165	212	0.4	0.3979	182	223	36	2	3
3	42	1	0	0	2	0	1	0	318	381	1	0	206	382	1	0	245	360	26	5	6
4	49	1	0		2	1	0.8	0.0969	294	357	1	0	272	376	1	0	199	290	12	5	5
5	63	1	1	1	2	0	0.32	0.4949	485	409	0.25	0.6021	188	303	0.2	0.699	347	319	19	3	5
6	53	2	1	0	1	1	0.4	0.3979	296	538	0.5	0.301	192	672	0.25	0.6021	254	575	28		15
7	56	1	0		2	0	0.32	0.4949	352	412	0.5	0.301	217	394	1	0	225	413	13	6	6
8	52	1	0	0	2	1	0.32	0.4949	266	352	0.32	0.4949	217	343	0.32	0.4949	253	239	30	3	8
9	41	1	0		2	0	1	0	287	434	1	0	256	466	1	0	282	378	23	1	1
10	49	1	0		2	0	0.32	0.4949	249	321	1	0	241	330	1	0	231	306	3	1	1
11	37	1	0	1	2	0	1	0	537	400	1	0	193	371	1	0	207	384	8	1	2
12	50	1	0	0	2	1	0.63	0.2007	406	383	0.63	0.2007	191	377	1	0	232	373	6	1	2
13	52	1	1	0	1	1	0.13	0.9031	542	490	0.25	0.6021	258	454	0.2	0.699	427	450	6		3
14	44	1	0		2	0	0.5	0.301	245	280	0.63	0.2007	233	285	0.5	0.301	232	286	10	4	4
15	57	1	1	1	2	0	0.5	0.301	393	426	0.5	0.301	199	393	0.8	0.0969	229	383	8	2	2
16	45	1	0	1	2	1	0.2	0.699	454	577	0.2	0.699	274	582	0.13	0.9031	304	659	14	5	7
17	48	1	0	1	2	1	0.5	0.301	357	363	0.63	0.2007	262	425	0.63	0.2007	81	361	13	3	4
18	37	1	0	0	1	0	0.05	1.301	387	380	0.05	1.301	422	455	0.02	1.699	542	404	23		1
19	49	1	0	1	2	0	0.32	0.4949	313	278	0.63	0.2007	122	261	0.25	0.6021	145	248	51	3	10
20	43	2	0	0	2	1	1	0	439	474	1	0	532	485	0.32	0.4949	362	394	40	5	12
21	43	2	0	0	2	1	0.25	0.6021	227	408	0.4	0.3979	329	401	0.25	0.6021	271	375	50	8	24
22	50	1	0	1	2	1	1	0	556	443	1	0	262	425	0.8	0.0969	197	354	15	4	6
23	43	1	0		2	0	0.63	0.2007	587	461	0.63	0.2007	362	202	0.63	0.2007	211	524	7	2	2
24	60	1	1		1	0	0.5	0.301	270	530	0.5	0.301	225	498	0.63	0.2007	267	516	6		1
25	45	1	0	1	2	0	1	0	369	526	1	0	255	358	1	0	367	558	7	1	2
26	51	2	0	0	2	0	0.5	0.301	311	274	0.63	0.2007	136	269	0.25	0.6021	161	243	50	2	5
27	58	2	0	0	2	1	0.8	0.0969	538	531	0.5	0.301	430	522	0	0.3979	176	448	6	3	3
28	51	1	0	1	2	1	0.2	0.699	441	474	0.5	0.301	145	384	0.9	0.0458	184	384	16	1	3
29	51	1	0	1	1	0	1	0	220	564	1	0	201	654	0.5	0.301	426	616	22		5
30	46	2	0	0	2	1	1	0	220	498	1	0	188	406	1	0	216	329	42	2	4
31	43	1	0	0	2	0	1	0	314	523	1	0	176	526	1	0	191	494	53	2	20
32	71	1	0	0	2	1	0.25	0.6021	417	363	0.63	0.2007	202	386	0.5	0.301	187	278	3	1	1
33	67	1	1	0	1	0	0.32	0.4949	290	218	0.2	0.699	352	225	0.2	0.699	354	237	4		3
34	62	2	0	0	2	0	0.25	0.6021	320	360	0.4	0.3979	228	351	0.4	0.3979	228	351	3	2	2
35	54	1	0	1	2	0	1	0	466	251	1	0	229	227	1	0	265	234	5	1	1
36	45	1	0		2	1	0.5	0.301	284	351	0.5	0.301	266	320	1	0	225	377	4	1	1
37	62	1	0		1	1	0.16	0.7959	728	238	0.32	0.4949	186	261	0.2	0.699	277	243	3		4
38	65	1	0	0	2	0	0.63	0.2007	485	363	1	0	278	369	1	0	369	349	13	5	5
39	45	1	0	1	2	0	0.4	0.3979	351	407	0.4	0.3979	186	437	0.4	0.3979	186	437	2	1	1
40	42	1	0	1	2	0	0.5	0.301	194	642	0.25	0.6021	194	642	0.5	0.301	222	600	10	4	6
41	46	2	0	0	2	1	0.2	0.699	773	625	0.2	0.699	630		1	0	224	507	5	3	3
42	40	1	1		1	1	1	0	297	424	1	0	288	432	1	0	367	485	7		5
43	60	2	0	0	1	1	0.4	0.3979	353	263	0.63	0.2007	362	262	0.63	0.2007	314	253	5		5
44	47	1	0	1	2	1	0.8	0.0969	506	383	1	0	251	400	1	0	287	439	5	3	3
45	60	1	0		2	1	0.25	0.6021	378	324	0.4	0.3979	252	274	0.32	0.4949	244	231	9	1	3
46	57	2	0		2	0	0.32	0.4949	341	372	1	0	197	362	1	0	197	362	1	1	1

47	39	1	0	0	2	0	1	0	528	363	0.8	0.0969	333	386	1	0	237	372	3	2	2
48	58	1	0	0	2	0	0.5	0.301	264	352	0.63	0.2007	165	360	1	0	163	348	4	1	1
49	57	1	1		2	0	0.5	0.301	286	461	0.5	0.301	211	462	0.5	0.301	211	457	3	1	2
50	47	1	1	0	1	1	0.4	0.3979	355	299	0.63	0.2007	316	303	1	0	266	303	2		2
51	46	1	0	1	1	1	0.25	0.6021	109	118	0.63	0.2007	298	102	0.63	0.2007	298	102	2		1
52	65	2	1		1	1	0.63	0.2007	280	259	0.63	0.2007	307	250	1	0	324	358	5		3
53	38	1	0	1	2	1	0.32	0.4949	256	327	0.32	0.4949	233	281	0.25	0.6021	262	279	4	1	1
54	57	1	0	0	2	0	0.5	0.301	501	270	0.63	0.2007	454	284	1	0	241	237	20	3	3
55	55	1	0	1	1	0	0.63	0.2007	467	292	1	0	255	280	1	0	260	250	1		1
56	35	2	0	0	2	1	0.2	0.699	272	532	0.13	0.9031	278	625	0.32	0.4949	186	185	4	2	2
57	71	2	1	0	1	1	0.5	0.301	438	177	0.32	0.4949	544	179	0.2	0.699	476	458	8		4
58	45	1	0	1	2	1	0.5	0.301	475	296	1	0	333	305	1	0	254	278	11	1	1
59	47	2	0		2	0	1	0	405	265	1	0	251	218	1	0	217	211	52	2	9
60	65	1	0		2	1	0.8	0.0969	470	483	1	0	246	497	0.63	0.2007	226	227	26	1	5
61	44	1	0		1	1	1	0	512	439	1	0	292	463	1	0	292	292	2		1
62	30	1	1	0	2	1	1	0	392	514	1	0	221	447	1	0	220	221	2	1	1
63	54	1	0	1	2	1	1	0	443	190	1	0	312	191	1	0	255	253	51	6	6
64	53	1	1		2	0	1	0	488	392	1	0	369	391	1	0	512	509	11	5	6
65	57	2	1	0	2	1	0.25	0.6021	293	531	0.32	0.4949	304	570	0.32	0.4949	217	219	3	2	2
66	46	1	0	1	2	0	0.25	0.6021	157	519	0.32	0.4949	143	404	0.25	0.6021	157	168	18	1	1
67	53	1	0	1	2	1	0.32	0.4949	581	456	0.25	0.6021	412	442	0.63	0.2007	237	237	8	2	2
68	42	2	0	0	2	1	1	0	399	486	1	0	223	480	1	0	236	234	6	2	2
69	44	1	0	0	2	0	1	0	329	517	1	0	274	477	1	0	276	271	2	1	1
70	42	1	0	0	2	1	0.25	0.6021	493	515	0.5	0.301	268	496	1	0	235	238	7	2	2
71	36	1	0		2	1	0.5	0.301	439	441	0.5	0.301	251	409	0.5	0.301	256	424	7	1	2
72	46	1	0		2	1	0.5	0.301	411	186	0.4	0.3979	418	169	0.5	0.301	293	174	8	3	3
73	47	2	0	0	2	0	0.5	0.301	313	329	0.32	0.4949	217	341	1	0	210	292	27	1	1
74	64	1	0	1	1	1	0.2	0.699	441	233	0.2	0.699	416	202	0.63	0.2007	380	190	4		3
75	57	1	0		2	1	0.25	0.6021	332	280	0.5	0.301	213	242	0.5	0.301	215	247	3	1	1
76	47	1	0	0	1	1	1	0	334	262	1	0	283	247	1	0	283	247	7		5
77	51	1	0	0	2	1	0.8	0.0969	427	420	0.63	0.2007	303	485	0.5	0.301	267	516	5	3	3
78	58	1	0		1	0	1	0	455	328	1	0	466	312	1	0	339	306	3		3
